# Exploring Drought Resistance Genes from the Roots of the Wheat Cultivar Yunhan1818

**DOI:** 10.3390/ijms252413458

**Published:** 2024-12-16

**Authors:** Linyi Qiao, Lifang Chang, Mengxiang Kai, Xueqi Zhang, Tingting Kang, Lijuan Wu, Xiaojun Zhang, Xin Li, Jiajia Zhao, Zhiyong Zhao, Jun Zheng

**Affiliations:** 1 College of Agronomy, Shanxi Key Laboratory of Crop Genetics and Molecular Improvement, Shanxi Agricultural University, Taiyuan 030031, China; linyi.qiao@sxau.edu.cn (L.Q.);; 2 Institute of Wheat Research, Shanxi Agricultural University, Linfen 041000, China; 3 Institute of Cotton Research, Shanxi Agricultural University, Yuncheng 044000, China

**Keywords:** wheat cv. YH1818, root, drought stress, WGCNA, candidate genes

## Abstract

The root is an important organ by which plants directly sense variation in soil moisture. The discovery of drought stress-responsive genes in roots is very important for the improvement of drought tolerance in wheat varieties via molecular approaches. In this study, transcriptome sequencing was conducted on the roots of drought-tolerant wheat cultivar YH1818 seedlings at 0, 2, and 7 days after treatment (DAT). Based on a weighted gene correlation network analysis of differentially expressed genes (DEGs), 14 coexpression modules were identified, of which five modules comprising 3107 DEGs were related to 2 or 7 DAT under drought stress conditions. A total of 223,357 single-nucleotide polymorphisms (SNPs) of these DEGs were retrieved from public databases. Using the R language package and GAPIT program, association analysis was performed between the 223,357 SNPs and the drought tolerance coefficient (DTC) values of six drought resistance-related traits in 114 wheat germplasms. The results revealed that 18 high-confidence SNPs of 10 DEGs, including *TaPK*, *TaRFP*, *TaMCO*, *TaPOD*, *TaC3H-ZF*, *TaGRP*, *TaDHODH*, *TaPPDK*, *TaLectin*, and *TaARF7-A*, were associated with drought tolerance. The RT–qPCR results confirmed that these genes were significantly upregulated by drought stress at 7 DAT. Among them, *TaARF7-A* contained three DTC-related SNPs, which presented two haplotypes in the tested wheat germplasms. YH1818 belongs to the Hap1 allele, which is involved in increased drought tolerance. This study revealed key modules and candidate genes for understanding the drought-stress response mechanism in wheat roots.

## 1. Introduction

In the context of climate change, water scarcity, and the rapid expansion of the global population, drought has become one of the most severe natural disasters limiting agricultural production [1]. Wheat (*Triticum aestivum* L.) is the most widely planted crop in the world, with numerous production areas located in arid or semiarid regions [2]. Exploring the response patterns of plants to drought stress and improving the adaptability of wheat varieties to arid environments are very important for ensuring food security.

In recent decades, advances in wheat genetics and physiology have shown that the epidermal wax on the aboveground parts of plants can improve the drought tolerance of wheat [3,4]. Furthermore, under drought stress conditions, the aboveground plant parts increase leaf stomatal closure through the phytohormone abscisic acid (ABA) signaling pathway, thereby reducing water loss caused by transpiration [5,6]. Several key drought tolerance-related factors that regulate the transcriptional response to ABA have also been identified, including PYRABACTIN RESISTANCE 1-LIKE (PYL), TYPE 2C PROTEIN PHOSPHATASE (PP2C), SUCROSE NON-FERMENTING 1-RELATED PROTEIN KINASE 2 (SnRK2), and ABA-RESPONSIVE ELEMENT-BINDING FACTOR (AREB/ABF) [6,7].

Compared with aboveground parts, roots are important organs through which plants directly sense variation in soil moisture. An ideal root system architecture can help wheat sufficiently absorb water from the soil and transport it to the aboveground parts, ensuring the normal growth of the plant under drought conditions [2]. Therefore, an in-depth exploration of drought stress-responsive genes in roots of tolerant germplasms can lay a theoretical foundation for the breeding of drought-resistant wheat. In Jinmai47, a well-known drought-resistant cultivar (cv.) in the North China Plain, 2911 differentially expressed genes (DEGs) in the roots were identified by comparing the transcriptomes of seedlings in a polyethylene glycol (PEG)-treated group and a control group; the DEGs that participated in the α-linolenic acid metabolism pathways were considered candidate genes related to drought resistance [8]. Similar analyses of cv. Jimai262 revealed that 9633 and 12,584 genes were differentially expressed in the roots 6 h and 48 h after PEG treatment, respectively, and candidate genes associated with reactive oxygen species (ROS) scavenging, the deposition of local lignin and suberin, and the high accumulation of osmoprotectants were further validated [9]. In addition, the RNA sequencing (RNA-Seq) of wheat roots in field environments revealed many drought stress-responsive genes from tolerant germplasms, such as the Indian variety NI5439 [10], Iranian landrace L-82 [11], Brazilian wheat line Abura [12], and the Chinese cultivars Luyuan502 [13] and Zhengmai366 [14].

Recent studies have confirmed the functions of several drought-induced candidate genes. For example, RNA-Seq results for the roots of a drought-tolerant Agropyron-wheat translocation line revealed that the wheat allele of the *LATERAL ROOT DENSITY* (*LRD*) gene is upregulated by water deficit stress in contrast with the Agropyron *LRD* allele, which is downregulated in response to water limitation. The suppression of *LRD* expression in wheat plants by RNAi conferred the ability to maintain root growth under water limitation conditions and had a positive pleiotropic effect on grain size and number. Further studies revealed that gibberellic acid (GA) promotes lateral root growth by regulating *LRD* under water stress [15]. Moreover, the transcription of a *SINA-type E3 UBIQUITIN LIGASE* gene, *TaSINA2B*, is highly induced by drought treatment. Variation in the *TaSINA2B* promoter contributes to drought stress regulation, whereas the TaSINA2B and TaSINA1D interaction positively regulates drought tolerance by promoting root growth [16].

Yunhan1818 (YH1818), approved by the National Crop Variety Approval Committee of China in 2024 (Approval No. 20241060), is a new drought-resistant and water-saving wheat cultivar bred at the Institute of Cotton Research, Shanxi Agricultural University. It has a developed root system and can grow in arid environments with good yield. Here, we performed RNA-Seq on the roots of YH1818 seedlings under drought stress and control conditions and used weighted gene coexpression network analysis (WGCNA) to identify drought stress-responsive modules. Association analysis was subsequently conducted on the basis of root drought tolerance coefficient (DTC) data from a set of wheat germplasms to further identify candidate genes associated with drought resistance.

## 2. Results

### 2.1. Transcriptional Response to Drought Stress in Wheat Roots

The transcriptome sequencing of root samples from the drought-resistant cv. YH1818 resulted in 178.03 Gb of clean reads. Each sample yielded clean data above 10.20 Gb, with Q30-based percentages greater than 93.36% and GC percentages ranging from 49.98 to 53.17% (Table S1); these results indicate that the RNA-Seq data were of high quality for further analysis. Subsequently, 1.06 Gb of the clean reads were mapped to the Chinese Spring reference genome RefSeq v1.0, and the uni-transcripts were annotated as known genes. A total of 54,053 genes with an average fragments per kilobase of exon model per million mapped fragments (FPKM) of >1 in at least one treatment were considered as expressed genes.

**Figure 1:**
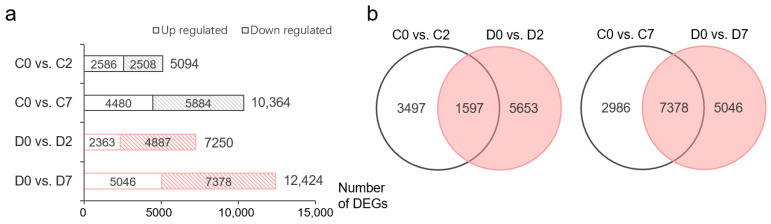
DEGs under drought stress conditions in the roots of wheat cv. YH1818. (**a**) Number of DEGs after 20% PEG treatment and control. (**b**) Venn diagram of DEGs. C0, C2, C7: control (1/2 Hoagland’s culture mixture) at 0, 2, and 7 DAT; D0, D2, D7: drought stress (1/2 Hoagland’s culture mixture with 20% PEG-6000) at 0, 2, and 7 DAT, marked in red.

Differential expression analysis revealed 18,491 DEGs that were upregulated or downregulated in at least one treatment, of which 7250 (2363 upregulated/4887 downregulated) and 12,424 (5046 upregulated/7378 downregulated) DEGs were identified at 2 and 7 days after treatment (DAT), respectively, compared with the transcriptome data at 0 DAT. Compared with those in the 0 DAT group, there were 5094 and 10,364 DEGs in the control treatment group at 2 and 7 DAT, respectively (Figure 1a). Furthermore, compared with those in the control treatment group, 5653 and 5046 DEGs only appeared in the drought treatment group at 2 and 7 DAT, respectively (Figure 1b).

### 2.2. Coexpression Modules of Drought-Stress-Responsive Genes

**Figure 2:**
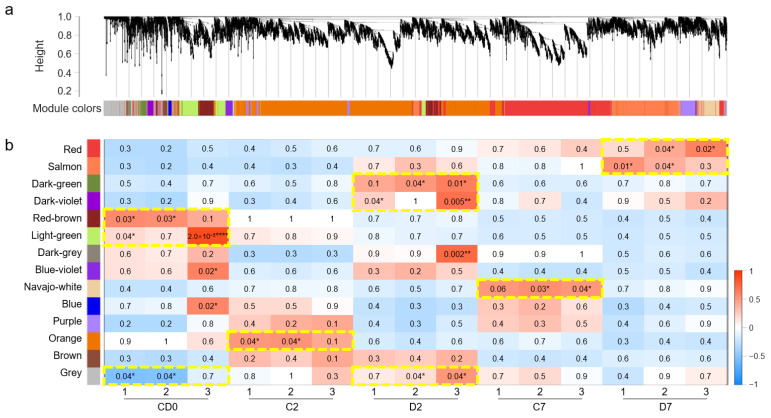
WGCNA for DEGs responding to drought stress in wheat roots. (**a**) Cluster dendrogram and module colors of 8597 DEGs. (**b**) Heatmap of the relationships between the modules and drought treatment. Positive and negative correlations are represented in red and blue, respectively. Each cell lists the *p* value to indicate the significance of the correlation, and the cells with significant correlations are marked with yellow dashed boxes; * indicates *p* < 0.05, ** indicates *p* < 0.01, and **** indicates *p* < 0.0001 according to the Pearson test. C2, C7: control at 2 and 7 DAT; D2, D7: drought stress at 2 and 7 DAT; CD0: control or drought stress at 0 DAT.

WGCNA was performed on the 18,491 DEGs screened above, and 8597 genes were clustered and divided into 14 modules on the basis of their expression patterns; each module was assigned a unique color (Figure 2a, Table S2). Among them, the Orange module contained the most DEGs (*n* = 3381), whereas the Blue module contained the fewest DEGs (*n* = 43). A correlation analysis revealed that the expression patterns of nine modules were significantly correlated with time points or treatments: (1) the Red-brown and Light-green modules were significantly positively correlated with 0 DAT; (2) the Orange module and Navajo-white module were significantly positively correlated with 2 and 7 DAT in the control treatment group, respectively; (3) the Dark-green and Dark-violet modules were significantly positively correlated with 2 DAT under drought stress; (4) the Red and Salmon modules were significantly positively correlated with 7 DAT under drought stress; and (5) the Gray module was significantly negatively correlated with 0 DAT but positively correlated with 2 DAT under drought stress (Figure 2b, Table S3).

### 2.3. Association Analysis Between DEGs and DTC

**Figure 3:**
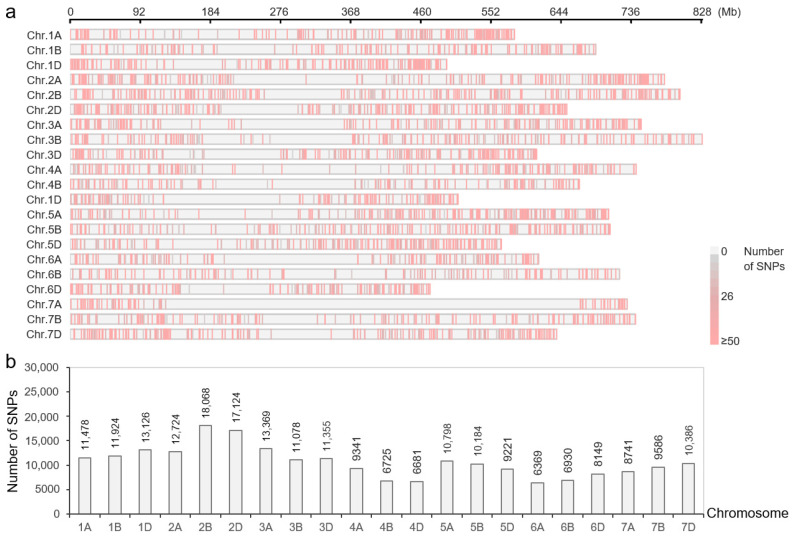
SNP loci of DEGs in drought-responsive modules. (**a**) Distribution of SNPs on wheat chromosomes. (**b**) The number of SNPs on each chromosome.

We focused on the Dark-green, Dark-violet, Red, Salmon, and Gray modules, which were correlated with 2 or 7 DAT under drought stress. The 3107 DEGs contained in these five modules were submitted to the WheatUnion database, and a total of 223,357 single-nucleotide polymorphisms (SNPs) distributed on 21 wheat chromosomes were identified from 114 wheat germplasms (Figure 3a). Among them, chromosome 2B had the most (18,068) SNPs, whereas chromosome 4B had the least (6725) SNPs (Figure 3b).

**Figure 4:**
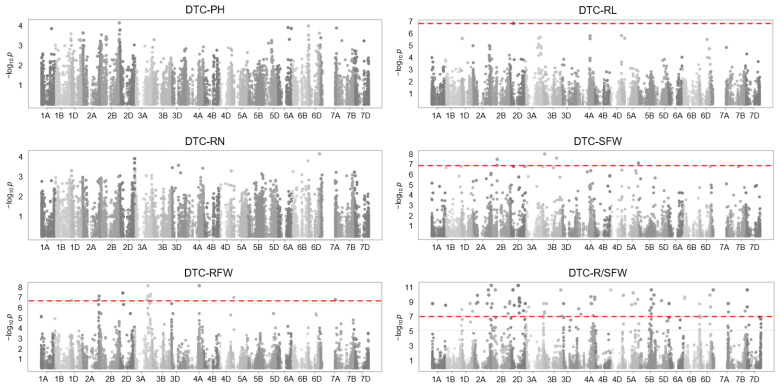
Manhattan plot of association analysis between SNPs of drought stress-response module genes and drought-tolerant phenotypes of 114 wheat germplasms. The threshold is set to −log_10_ *p* > 7. DTC: drought tolerance coefficient; PH: plant height; RL: root length; RN: root number; SFW: shoot fresh weight; RFW: root fresh weight; R/SFW: the ratio of RFW/SFW.

An association analysis was conducted between these 223,357 SNPs and the data of six phenotypes related to the DTC of 114 wheat germplasms, and the results revealed that no SNP was correlated with the DTC of plant height (PH) and root number (RN), whereas one SNP was correlated with the DTC of root length (RL); six SNPs were correlated with the DTC of shoot fresh weight (SFW), 11 SNPs were correlated with the DTC of root fresh weight (RFW), and 69 SNPs were correlated with the DTC of the ratio of RFW/SFW (R/SFW) (−log_10_ *p* > 7) (Figure 4).

### 2.4. DEGs That Responding to Drought Stress

After further screening, 18 high-confidence SNPs of 10 DEGs were selected, with a minimum allele frequency of 0.1 and a maximum missing rate of 0.05 (Table 1). Among them, one SNP of *TraesCS2D02G201400* was associated with DTC-RL; three SNPs of *TraesCS2B02G244000* and *TraesCS3A02G537700* were associated with DTC-SFW; five SNPs of *TraesCS1D02G053700*, *TraesCS2A02G547800*, *TraesCS3A02G325100*, and *TraesCS5A02G097300* were associated with DTC-RFW; and the nine SNPs of *TraesCS1A02G043900*, *TraesCS2A02G547800*, *TraesCS3A02G008900*, and *TraesCS7A02G556200* were associated with DTC-R/SFW.

Interestingly, all 10 DEGs belonging to the Red and Salmon modules were significantly positively correlated with 7 DAT under drought stress (Table 1). Among them, five genes encode enzymes, including *TraesCS2B02G244000* that encodes kinase/pyrophosphorylase (PPDK), *TraesCS5A02G097300* that encodes dihydroorotate dehydrogenase (DHODH), *TraesCS1A02G043900* that encodes multicopper oxidase (MCO), *TraesCS3A02G008900* that encodes protein kinase (PK), and *TraesCS3A02G325100* that encodes peroxidase (POD); the remaining five genes encode different types of proteins, including *TraesCS7A02G556200* that encodes a ring finger protein (RFP), *TraesCS3A02G537700* that encodes a zinc finger CCCH domain protein (C3H-ZF), *TraesCS1D02G053700* that encodes glycine-rich protein (GRP), *TraesCS2D02G201400* that encodes a legume lectin domain, and *TraesCS2A02G547800* that encodes an auxin response factor (ARF).

**Figure 5:**
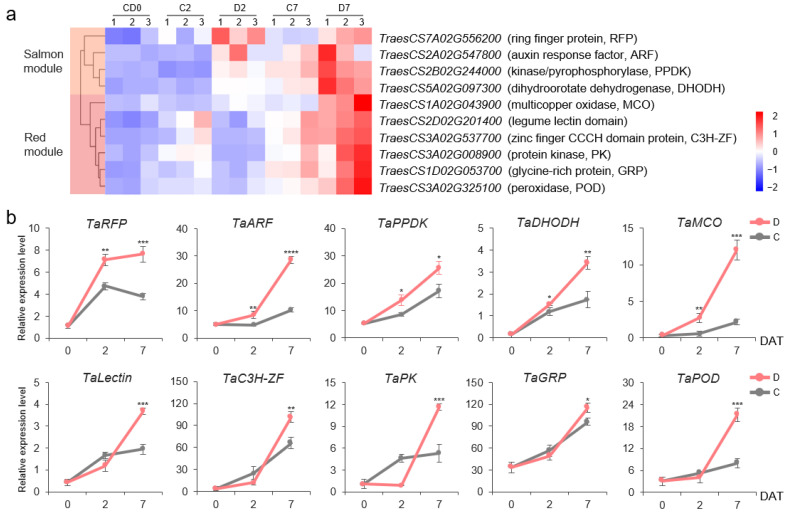
Response of candidate genes to drought stress in the roots of wheat cv. YH1818. (**a**) Cluster diagram of the transcription data of 10 DEGs after PEG treatment. FPKM values were normalized by z-score to present different degrees of upregulation, with small upregulation changes appearing in blue and large upregulation changes appearing in red. (**b**) RT–qPCR results of 10 DEGs after drought stress. D: drought treatment; C: control. The bars indicate the standard error. * indicates *p* < 0.05, ** indicates *p* < 0.01, *** indicates *p* < 0.001, and **** indicates *p* < 0.0001 according to the *t* test.

The RNA-Seq results of the YH1818 roots revealed that the expression patterns of these 10 DEGs were clustered into two modules; four genes belonged to the Salmon module and the other including six genes belonged to the Red module. The transcription levels of the *TaRFP*, *TaARF*, *TaPPDK*, and *TaDHODH* genes in the Salmon module at 2 and 7 DAT under drought treatment were significantly greater than those under the control treatment, whereas the *TaMCO*, *TaLectin*, *TaC3H-ZF*, *TaPK*, *TaGRP*, and *TaPOD* genes in the Red module were significantly upregulated only at 7 DAT under drought treatment (Figure 5a, Table S4). RT–qPCR confirmed these results, indicating that these 10 DEGs are drought stress-responsive genes (Figure 5b).

### 2.5. KASP Marker Validation of Candidate Gene TaARF7-A

**Figure 6:**
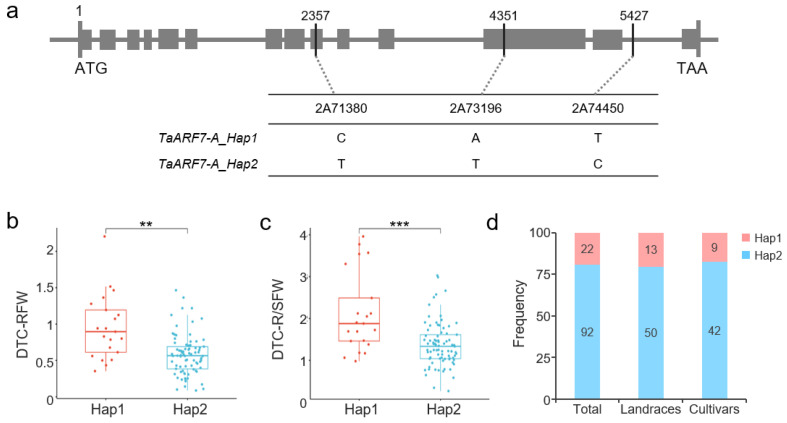
Two haplotypes of *TaARF7-A*. (**a**) Location of three SNPs 2A71380, 2A73196, and 2A74450 in the gene. Gray boxes indicate exons. (**b**,**c**) Phenotypic differences in DTC-RFW and DTC-R/SFW between Hap1 and Hap2. ** indicates *p* < 0.01 and *** indicates *p* < 0.001 according to a *t* test. (**d**) Distribution frequency of the two haplotypes in 114 wheat germplasms, including 63 landraces and 51 cultivars.

Among the 10 drought stress-responsive genes, *TraesCS2A02G547800*, which encodes ARF, was significantly correlated with both DTC-RFW and DTC-R/SFW. According to the wheat ARF family number [17], this gene was named *TaARF7-A*. *TaARF7-A* contains three SNPs associated with DTCs: 2A71380 [T/C], located in exon 10 of *TaARF7-A*, increasing DTC-R/SFW (*p* = 1.25 × 10^−11^) and DTC-RFW (*p* = 2.36 × 10^−7^); 2A73196 [T/A], located in exon 13, increasing DTC-R/SFW (*p* = 1.07 × 10^−8^); and 2A74450 [C/T], located in intron 14, increasing DTC-R/SFW (*p* = 2.33 × 10^−8^) (Table 1, Figure 6a).

These three SNPs displayed two haplotypes, Hap1 and Hap2, in the 114 wheat germplasms used in this study. We transformed 2A71380 and 2A73196 into Kompetitive Allele-Specific PCR (KASP) markers for confirmation and obtained the expected typing results; the results revealed that Hap1 had a higher DTC-RFW (*p* = 0.0016) and DTC-R/SFW (*p* = 0.0004) than Hap2 (Figure 6b,c), whereas there was no significant difference in the other DTCs, such as PH, RL, RN, and SFW (Figure S1). These results indicated that Hap1 may improve plant drought tolerance by increasing root biomass.

Further analysis revealed that the distribution frequency of *TaARF7-A_Hap1* was low in the 114 wheat germplasms (19.30%) (Figure 6d, Table S5). Among them, the frequency of Hap1 in cultivars (17.65%, *n* = 51) was lower than that in landraces (20.63%, *n* = 63), indicating that the drought resistance of modern varieties may have decreased, accompanied by the application of irrigation facilities and improvements in cultivation techniques.

## 3. Discussion

### 3.1. Modules That Responding Drought Stress

Root system architecture and root biomass affect the adaptability of plants to external drought environments [2]. Research on model plants has shown that some transcription factors are generally induced by drought stress to regulate hormone signaling pathways such as the ABA and auxin pathways. As a result, the activity of enzymes related to physiological and biochemical reactions and oxidative repair is increased, and large amounts of organic compounds such as lignin, sucrose, and proline are synthesized to thicken the cell wall, increase the cell osmotic pressure, and alleviate damage caused by stress [18,19].

Exploring key drought resistance modules and candidates that regulate these processes in wheat germplasm not only helps to increase our understanding of the mechanisms underlying the stress response but also identifies new genes and markers for molecular breeding. There have been reports on the discovery of genes in several Chinese drought-tolerant wheat cultivars, such as Jimai 262, Jinmai 47, and Luyuan 502 [8,13,14]. YH1818 is a new wheat cv. with excellent drought resistance. From the RNA-Seq results of YH1818 roots, we identified two coexpression modules, Red and Salmon, related to 7 DAT with PEG, containing 2094 and 722 DEGs, respectively. The Kyoto Encyclopedia of Genes and Genomes (KEGG) enrichment results revealed that the DEGs in these two modules were associated with three main categories: plant hormone signal transduction, phenylpropanoid biosynthesis, and starch and sucrose metabolism (Figure S2). The phenylpropanoid pathway provides precursors for lignin biosynthesis [20]. The WGCNA and enrichment results revealed that the Red and Salmon modules were highly correlated with the drought stress response.

### 3.2. Candidate Genes in Red Module and Salmon Module

Through association analysis, 10 candidate genes related to drought tolerance, including *TaPK*, *TaRFP*, *TaMCO*, *TaPOD*, *TaC3H-ZF*, *TaGRP*, *TaDHODH*, *TaPPDK*, *TaLectin*, and *TaARF7-A*, were identified in the Red and Salmon modules.

It is well known that PKs are central components in the response of plants to abiotic stresses. Sucrose non-fermenting (SNF1)-related protein kinase 2s (SnRK2s) are central components, while the mitogen-activated protein kinase kinase kinase (MAPKKK)-mediated signaling cascade also plays important roles in plant responses to drought stress [21,22]. In wheat, several drought-responsive genes that encode serine/threonine protein kinases, such as *TaSnRK2.4* [23] and *Td4IN2* [24], were identified. The candidate gene *TaPK* identified in our study may perform similar functions because it encodes a serine/threonine protein kinase. Moreover, RFP can interact with PKs. In Arabidopsis, the RING finger proteins RGLG1 and RGLG2 negatively modulate MAPKKK18-mediated drought stress tolerance [25].

MCOs and PODs are two key enzymes in the lignin biosynthesis pathway that play essential roles in root growth and stress resistance [26]. Laccases (LACs) are an important family of MCOs and are required for lignification in plants. Several *LAC* genes control drought resistance by regulating lignin deposition in roots, and they are targeted by *miR397* or *miR408* in wheat, rice, maize, cowpea, and chickpea [27,28,29]. In addition, POD and lignin-related cinnamyl alcohol dehydrogenase (CAD) interact with an AP2/ERF transcription factor related to ABI3/VP1 (RAV) to affect H_2_O_2_ and endogenous lignin accumulation, respectively, which are important in drought stress resistance [30].

C3H-ZFs directly regulate downstream genes related to drought stress at the transcription level [31]. In rice, transgenic lines of the C3H-ZF genes *OsC3H10* or *OsTZF5* exhibited increased drought resistance. The overexpression of *OsC3H10* increased the transcription level of stress response-related genes, including *LATE EMBRYOGENESIS ABUNDANT PROTEINs* (*LEAs*), *PATHOGENESIS-RELATED GENEs* (*PRs*), and *GERMIN-LIKE PROTEINs* (*GLPs*) [32]. Compared with the wild type, the *OsTZF5*-overexpressing lines had 609 upregulated genes and 196 downregulated genes and exhibited an increased survival rate under drought stress without growth retardation [33]. In addition, *OsGRP3* [34] and *OsDHODH1* [35], which encode the same products as *TaGRP* and *TaDHODH*, respectively, have also been reported to be associated with drought tolerance in rice.

### 3.3. TaARF7-A Is Involved Drought Tolerance

The auxin signaling pathway is an important hormone signaling pathway that responds to drought stress in plants and has significant implications for agricultural production. ARFs are the key components of auxin signaling, which links the nuclear auxin pathway and the transcriptional regulation of downstream genes [36,37]. In rice, OsARF23 represses the early-auxin-response gene *DEEPER ROOTING 1* (*DRO1*) to control the root growth angle in response to drought conditions [38]. Moreover, an increasing number of studies have identified numerous *ARF* genes related to drought resistance, including *IbARF5* in sweet potato [39], *MdARF17* in apple [40], and *SlARF2* in tomato [41].

In previous research [42], RT-qPCR analysis was performed to determine expression levels of *TaARF* family in 14-day-old seedlings of wheat cv. Jinghua9 treated with PEG-6000 for 0, 1, 2, 5, 10, or 24 h, respectively. The results showed that, within 1 h of treatment, 19 *TaARF* genes including *TaARF7* reached their highest expression levels, followed by a gradual decrease back to their expression levels at 0 h, which preliminarily revealed the early response of *TaARFs* to drought stress. Moreover, RAC875_c1706_1888, a SNP marker linked with the major QTL cluster for physiological and agronomical traits mapped under drought conditions [43], was located in the 3’-untranslated region of *TaARF7-A*. In this study, *TaARF7-A* was significantly upregulated by drought stress at 7 DAT in the roots of cv. YH1818. Among the tested wheat germplasms, *TaARF7-A* presented two haplotypes, and varieties carrying Hap1 had greater root biomass and better drought tolerance than those carrying Hap2. The distribution frequency of the favored *TaARF7-A _Hap1* in cultivars is very low, demonstrating good application prospects. Altogether, *TaARF7-A* was involved in drought stress response in different varieties, tissues, or treatment periods. We will verify the drought resistance phenotype of *TaARF7-A* and elucidate its molecular regulatory mechanism in future work.

## 4. Materials and Methods

### 4.1. Plant Materials and Drought Treatment

The drought-tolerant cultivar YH1818, bred by the Institute of Cotton Research (Yuncheng, China), Shanxi Agricultural University, was used for RNA-Seq and RT–qPCR. A set of wheat germplasms containing 114 varieties [44] was used for association analysis, marker verification and haplotype discrimination. These materials were provided by the Shanxi Key Laboratory of Crop Genetics and Molecular Improvement.

Seeds were germinated on Petri dishes with moist filter paper. Uniform seeds with approximately 3 cm long embryonic roots were selected and transferred to sterile plastic boxes containing 1/2 Hoagland’s culture mixture in a growth chamber under a 22/16 °C (day/night) temperature regime and a 16/8 h (light/dark) photoperiod with 60% relative humidity. When the seedlings grew to the three-leaf stage, they were exposed to 1/2 Hoagland’s culture mixture with 20% PEG-6000 for drought-stress treatment and without PEG-6000 as a control.

### 4.2. RNA-Seq

Three biological replicates of YH1818 seedling roots were collected at 0, 2, and 7 DAT and frozen in liquid nitrogen. These root samples were then sent to Biomarker Technologies Co., Ltd. (Beijing, China) to construct cDNA libraries, and transcriptome sequencing was performed on the HiSeq4000 platform (Illumina, San Diego, CA, USA). Clean reads were obtained by removing low-quality reads containing adapters and poly-N (>10%) or with a quality score <30 from the raw data and mapping them to the Chinese Spring reference genome (RefSeq v1.0, http://wheat-urgi.versailles.inra.fr/, accessed on 20 May 2024). The transcript level of each gene was measured with FPKM values calculated from the following formula:

FPKM = cDNA Fragments/Mapped Fragments (Millions) * Transcript Length(kb), where “cDNA Fragments” represents the number of fragments mapped to a certain transcript, “Mapped Fragments (Millions)” represents the total number of fragments mapped to the transcript, measured in units of 10^6^, and “Transcript Length (kb)” represents the length of the certain transcript, measured in units of 10^3^ bases. Genes with FPKM values below 1 in all samples were defined as nonexpressed genes. Differential expression analysis between the control and drought-stress groups was performed with the DESeq R package (version 1.10) [45], with thresholds of |log_2_FoldChange| ≥ 1 and FDR ≤ 0.01. For visualization, FPKM values were submitted to SRplot platform (www.bioinformatics.com.cn/SRplot, accessed on 21 May 2024) after z-score normalization to generate a heatmap.

### 4.3. WGCNA

WGCNA was performed with R (version 4.3.1) software, and the WGCNA package (version 1.72) was used to analyze the root RNA-Seq data as previously described [46]. In brief, the transcript levels of all DEGs with FPKMs ≥ 1 were converted into a similarity matrix and transformed into a topological overlap matrix. Genes with similar expression patterns were categorized into different modules with a bottom-up algorithm and a module minimum-size cutoff of 30. The correlation between module eigengenes and the treatments was calculated with a Pearson test, and the individual modules with *p* < 0.05 were considered significantly correlated with the treatment course. In addition, DEGs in the Red and Salmon modules were subjected to KEGG enrichment analysis with the BMKCloud platform (www.biocloud.net, accessed on 21 May 2024).

### 4.4. Association Analysis

The drought tolerance coefficient of 7-DAT-plants in each wheat germplasm was measured according to the following formula: DTC = PV_D_/PV_C_, where PV_D_ is the phenotypic value of the trait expressed under drought stress, and PV_C_ is the value obtained in the control treatment. Ten individual plants per germplasm were tested. Six drought resistance-related traits of 7-DAT plants were identified to calculate the DTC: plant height (PH) and shoot fresh weight (SFW) were measured for the aboveground part (shoot), root length (RL), root number (RN), and root fresh weight (RFW) were measured for the underground part (root), and R/SFW was the ratio of RFW to SFW. Information on biallelic SNPs in the gene region was downloaded from the WheatUnion database (http://wheat.cau.edu.cn/WheatUnion/, accessed on 24 June 2024). On the basis of 114 wheat germplasm resources, the correlation between the DTC data and DEGs was evaluated with association analysis in R (version 4.3.1) software with the GAPIT package (version 3) [47]. The correlation was considered significant when −log_10_ *p* > 7.

### 4.5. RT-qPCR

RT-qPCR was used to validate the transcription level of DEGs. The remaining root samples of YH1818 from the RNA-Seq project were used for RNA extraction using an RNA Extraction kit (Tianmo Bio, Beijing, China). The RNAs were reverse transcribed into cDNA using an RT Reagent kit (Takara Bio, Shiga, Japan). Quantitative PCR was performed using TB Green Taq II (Takara Bio, Shiga, Japan) with *ACTIN* as the endogenous control. Each sample was conducted with three technical replications. Information about the primers is listed in Table S6.

### 4.6. KASP

Total DNA was extracted for each of the 114 wheat germplasms using an improved cetyltrimethylammonium bromide (CTAB) method. KASP primers were designed based on selected SNPs (Table S6). The KASP reaction system consists of 10 μL of 2 × KASP Master Mix (Aijixi Technology, Shanghai, China) (each mix was 5.0 μL), a DNA template (30 ng/μL) 4.85 μL, and primer mixture (100 pmol/μL) 0.15 μL. The KASP reactions were performed on a QuantStudio 3 Real-time PCR System (Applied Biosystems, Carlsbad, CA, USA) by running the following program: denature at 94 °C for 10 min, 10 cycles of touch-down PCR (94 °C for 20 s; touchdown at 60 °C initially and decreasing by −0.6 °C per cycle for 60 s), and 40 additional cycles (94 °C for 20 s; 55 °C for 60 s). PCR products were calculated in a fluorescence scanner under FAM and HEX channels.

### 4.7. Statistical Analysis

The Origin software (version 3.1) was used to perform the statistical analysis on RT-qPCR data by one-way analysis of variance (ANOVA), and *p* < 0.05 was considered a statistically significant difference.

## 5. Conclusions

This study revealed two coexpression modules that highly correlated with drought stress-responsive genes in cv. YH1818 roots using RNA-seq and WGCNA and identified ten candidate genes related to drought resistance via association analysis and RT-qPCR. Our results will contribute to a further understanding of the drought stress response mechanism in plant roots and provide candidate genes for improving drought-tolerant wheat varieties.

## Figures and Tables

**Table 1:** SNPs that significantly correlated with DTC.

SNP-ID	Position (IWGSC v1.0)	Trait	*p*-Value	Gene	Module
2D01736	chr.2D:153001736	DTC-RL	1.55 × 10^−7^	*TraesCS2D02G201400*	Red
2B02916	chr.2B:248102916	DTC-SFW	1.42 × 10^−7^	*TraesCS2B02G244000*	Salmon
2B02985	chr.2B:248102985	DTC-SFW	3.76 × 10^−8^	*TraesCS2B02G244000*	Salmon
3A80441	chr.3A:749280441	DTC-SFW	1.81 × 10^−7^	*TraesCS3A02G537700*	Red
1D43275	chr.1D:34943275	DTC-RFW	1.39 × 10^−7^	*TraesCS1D02G053700*	Red
2A71380	chr.2A:755771380	DTC-RFW	2.36 × 10^−7^	*TraesCS2A02G547800*	Salmon
3A39906	chr.3A:569639906	DTC-RFW	5.32 × 10^−8^	*TraesCS3A02G325100*	Red
3A40640	chr.3A:569640640	DTC-RFW	5.04 × 10^−8^	*TraesCS3A02G325100*	Red
5A80484	chr.5A:138080484	DTC-RFW	6.72 × 10^−8^	*TraesCS5A02G097300*	Salmon
1A39018	chr.1A:24339018	DTC-R/SFW	3.13 × 10^−9^	*TraesCS1A02G043900*	Red
1A39019	chr.1A:24339019	DTC-R/SFW	3.13 × 10^−9^	*TraesCS1A02G043900*	Red
2A71380	chr.2A:755771380	DTC-R/SFW	1.25 × 10^−11^	*TraesCS2A02G547800*	Salmon
2A73196	chr.2A:755773196	DTC-R/SFW	1.07 × 10^−8^	*TraesCS2A02G547800*	Salmon
2A74450	chr.2A:755774450	DTC-R/SFW	2.33 × 10^−8^	*TraesCS2A02G547800*	Salmon
3A60070	chr.3A:8260070	DTC-R/SFW	1.93 × 10^−7^	*TraesCS3A02G008900*	Red
3A60135	chr.3A:8260135	DTC-R/SFW	3.49 × 10^−8^	*TraesCS3A02G008900*	Red
7A22098	chr.7A:727722098	DTC-R/SFW	3.07 × 10^−9^	*TraesCS7A02G556200*	Salmon
7A22246	chr.7A:727722246	DTC-R/SFW	3.90 × 10^−8^	*TraesCS7A02G556200*	Salmon

## Data Availability

Data are contained within the article and Supplementary Materials.

## Supplementary Materials

The following supporting information can be downloaded at https://www.mdpi.com/article/10.3390/ijms252413458/s1.
