# Supplementary material for: Exploring Drought Resistance Genes from the Roots of the Wheat Cultivar Yunhan1818
Source: Int J Mol Sci. 2024 Dec 16;25(24):13458. doi: 10.3390/ijms252413458 (PMC11679818; doi:10.3390/ijms252413458)

Figure S1. DTC values of PH, RL, RN and SFW between *TaARF7-A_Hap1* and *_Hap2*.

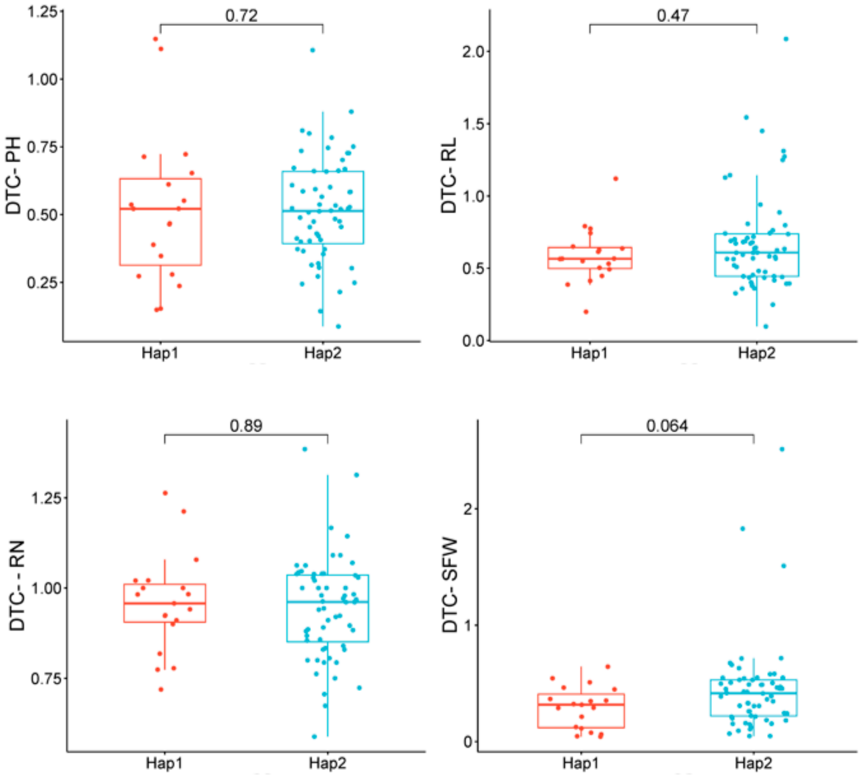

Figure S2. KEGG enrichment results of Red module and Salmon module.

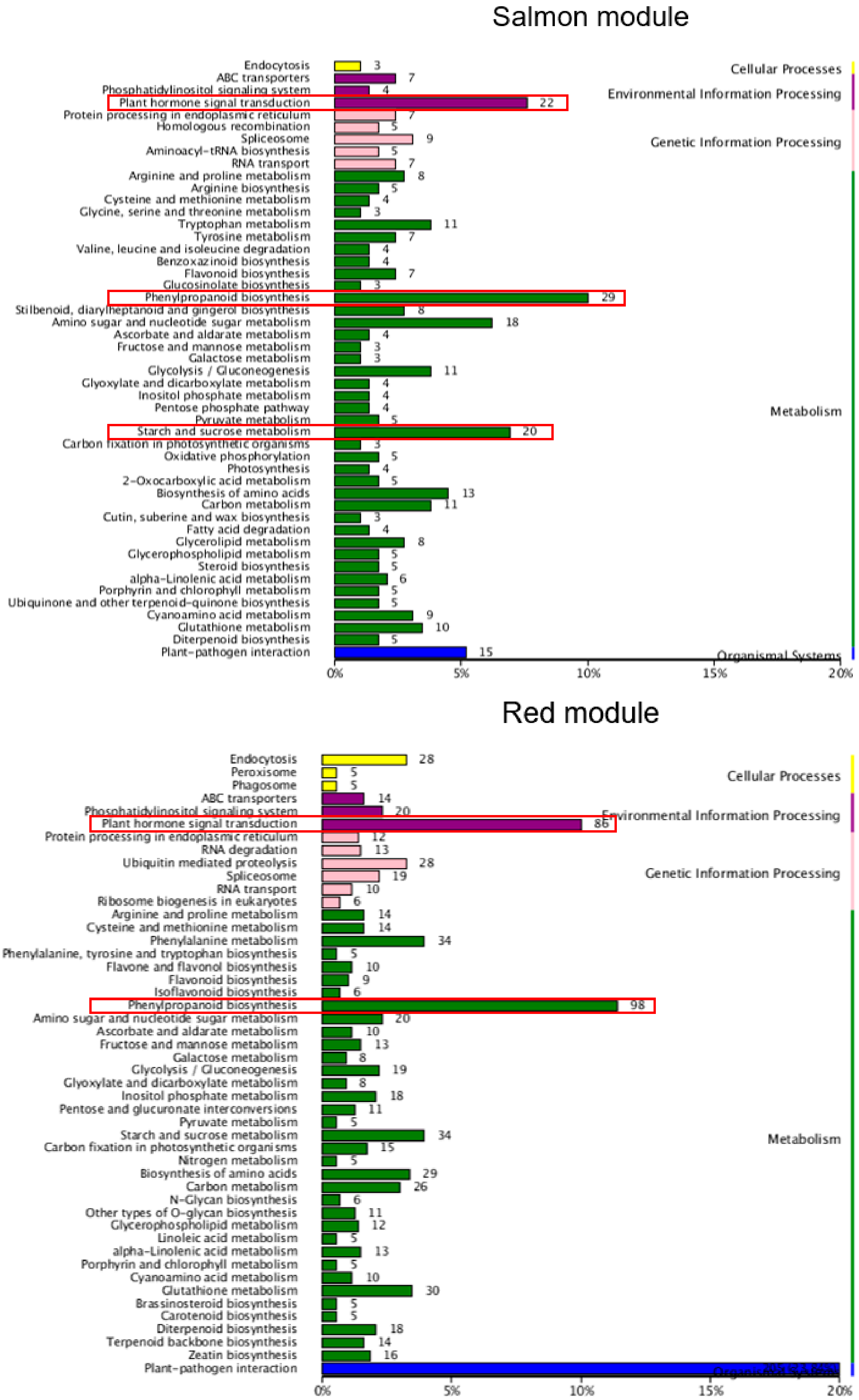

Supplement: Supplementary file 1 [file ijms-25-13458-s001.zip › Supplementary Figures S1-S2.pdf]
